# Identification and characterization of a novel chromosomal aminoglycoside 3’-*O*-phosphotransferase, APH(3′)-Id, from *Kluyvera intermedia* DW18 isolated from the sewage of an animal farm

**DOI:** 10.3389/fmicb.2023.1224464

**Published:** 2023-08-28

**Authors:** Yuning Sha, Naru Lin, Guozhi Zhang, Yuan Zhang, Jingxuan Zhao, Junwan Lu, Tingting Zhu, Xueya Zhang, Qiaoling Li, Hailin Zhang, Xi Lin, Kewei Li, Qiyu Bao, Dong Li

**Affiliations:** ^1^The Second Affiliated Hospital and Yuying Children’s Hospital, Wenzhou Medical University, Wenzhou, China; ^2^Key Laboratory of Medical Genetics of Zhejiang Province, Key Laboratory of Laboratory Medicine, Ministry of Education, China, School of Laboratory Medicine and Life Sciences, Wenzhou Medical University, Wenzhou, China; ^3^Medical Molecular Biology Laboratory, School of Medicine, Jinhua Polytechnic, Jinhua, China

**Keywords:** *Kluyvera intermedia*, resistance gene, APH(3′)-Id, aminoglycoside 3’-*O*-phosphotransferase, kinetic parameter

## Abstract

**Background:**

Aminoglycosides, as important clinical antimicrobials, are used as second-line drugs for treating multidrug-resistant tuberculosis or combined with β-lactam drugs for treating severe infections such as sepsis. Aminoglycoside-modifying enzyme (AME) is the most important mechanism of aminoglycoside resistance and deserves more attention.

**Methods:**

The bacterium *Kluyvera intermedia* DW18 was isolated from the sewage of an animal farm using the conventional method. The agar dilution method was used to determine the minimum inhibitory concentrations (MICs) of antimicrobials. A novel resistance gene was cloned, and the enzyme was expressed. The kinetic parameters were measured by a SpectraMax M5 multifunctional microplate reader. Bioinformatic analysis was performed to reveal the genetic context of the *aph(3′)-Id* gene and its phylogenetic relationship with other AMEs.

**Results:**

A novel aminoglycoside 3′-*O*-phosphotransferase gene designated *aph(3′)-Id* was identified in *K. intermedia* DW18 and shared the highest amino acid identity of 77.49% with the functionally characterized aminoglycoside 3′-*O*-phosphotransferase APH(3′)-Ia. The recombinant plasmid carrying the novel resistance gene (pMD19-*aph(3′)-Id*/*E. coli* DH5α) showed 1,024-, 512-, 128- and 16-fold increased MIC levels for kanamycin, ribostamycin, paromomycin and neomycin, respectively, compared with the reference strain DH5α. APH(3′)-Id showed the highest catalytic efficiency for ribostamycin [*k*_cat_*/K*_m_ of (4.96 ± 1.63) × 10^5^ M^−1^/s^−1^], followed by paromomycin [*k*_cat_*/K*_m_ of (2.18 ± 0.21) × 10^5^ M^−1^/s^−1^], neomycin [*k*_cat_*/K*_m_ of (1.73 ± 0.20) × 10^5^ M^−1^/s^−1^], and kanamycin [*k*_cat_*/K*_m_ of (1.10 ± 0.18) × 10^5^ M^−1^/s^−1^]. Three conserved functional domains of the aminoglycoside phosphotransferase family and ten amino acid residues responsible for the phosphorylation of kanamycin were found in the amino acid sequence of APH(3′)-Id. No mobile genetic element (MGE) was discovered surrounding the *aph(3′)-Id* gene.

**Conclusion:**

In this work, a novel aminoglycoside 3’-*O*-phosphotransferase gene designated *aph(3′)-Id* encoded in the chromosome of the environmental isolate *Kluyvera intermedia* DW18 was identified and characterized. These findings will help clinicians select effective antimicrobials to treat infections caused by pathogens with this kind of resistance gene.

## Introduction

The genus *Kluyvera* of the family Enterobacteriaceae was defined in 1981 (Farmer et al., 1981), and at present, it consists of 5 species, including *Kluyvera georgiana*, *Kluyvera ascorbata*, *Kluyvera cryocrescens*, *Kluyvera intermedia* and *Kluyvera sichuanensis*. As a rare pathogenic agent, *Kluyvera intermedia* has the potential to cause a variety of infections in humans, such as abdominal abscesses, septic shock, and soft tissue, urinary and bloodstream infections (Carter and Evans, 2005). In 2005, *Enterobacter intermedius* was included in *Kluyvera cochleae* of the genus *Kluyvera* and was subsequently officially named *Kluyvera intermedia* after biochemical, DNA hybridization and phylogenetic relationship analyses (Pavan et al., 2005). Besides isolation from human samples, which has certain clinical significance (Prats et al., 1987), strains of *K. intermedia* have mainly been isolated from surface water and unpolluted soil (Grimont and Grimont, 2006). *K. intermedia* has been reported to show resistance to some β-lactams (penicillin G, oxacillin, cefoxitin, cefepime and ceftriaxone) and carbapenems (imipenem and meropenem) and was naturally susceptible to most aminoglycosides, such as gentamicin, tobramycin, ribostamycin, spectinomycin and neomycin (Stock, 2002; Ribeiro et al., 2014; Paskova et al., 2018).

It has been recognized that the environment can act as an important source and transmission route of resistance genes (Martínez, 2008; Bengtsson-Palme et al., 2014). Long before antibiotics were used in the clinic, resistance genes were present in bacteria in the natural environment, as evidenced by the discovery of the vancomycin resistance element VanA in 30,000-year-old permafrost (D’Costa et al., 2011). This suggests that resistance genes exist and spread even without modern clinical antibiotic selection pressure. Globally, water and soil can act as very large reservoirs of resistance genes (Suzuki and Hoa, 2012; Schulz et al., 2017), and these resistance genes can be captured by mobile genetic elements (MGEs), such as integrons, transposons, or chromosomal islands, and introduced into clinical pathogens through horizontal transfer (Forsberg et al., 2012; Djordjevic et al., 2013).

Aminoglycosides are highly effective broad-spectrum antibiotics that are active against a variety of gram-positive and gram-negative organisms (Krause et al., 2016). They remain an important part of clinical antimicrobial treatment and can be combined with β-lactam drugs for treating severe sepsis (Dellinger et al., 2013) and second-line drugs for treating multidrug-resistant tuberculosis (Caminero and Scardigli, 2015). The mechanisms of aminoglycoside resistance include aminoglycoside-modifying enzyme (AME) (Ramirez and Tolmasky, 2010), 16S rRNA methylation (Doi and Arakawa, 2007) and efflux pump (Magnet et al., 2001), the most common among which is AME. In susceptible strains, aminoglycosides exert antibacterial effects in two steps: transmembrane entry into cells and interaction with ribosomes leading to protein synthesis errors. The situation differs in strains producing aminoglycoside-modifying enzymes (AMEs); aminoglycosides entering the cell are inactivated by the AMEs so that they cannot interact with ribosomes (Azucena and Mobashery, 2001). AMEs can inactivate aminoglycosides by making different types of modifications to them and are generally divided into three groups, including aminoglycoside acetyltransferases (AACs), aminoglycoside phosphotransferases (APHs) and aminoglycoside nucleotidyltransferases (ANTs) (Azucena and Mobashery, 2001). The designations are composed of the enzyme type and enzyme modification site, with Roman numerals representing unique resistance and lowercase letters representing unique protein names (Shaw et al., 1993).

APHs can catalyze the transfer of a phosphate group from ATP or GTP to the hydroxyl group of aminoglycoside molecules, thus preventing the drug from binding to the aminoacyl-tRNA site (A site) in the prokaryotic 30S ribosomal subunit (Tsai et al., 2013; Costa et al., 2020). It was found that APHs shared similar active sites with eukaryotic protein kinases (ePKs) and common ePK-like folds in the tertiary structure, and the inhibitors of ePKs also showed inhibitory activity against APHs, which suggested that they might have been derived from a common ancestor (Hon et al., 1997; Stogios et al., 2013). Some APHs were also found to have NTPs hydrolase activity and can hydrolyze NTPs in the absence of an antibiotic substrate, but the efficiency of NTP hydrolysis was far less than the catalytic efficiency of the antibiotic substrate, so it had little impact on the modification of antibiotics (Kim C. et al., 2006; Smith et al., 2019). The APH classes and subclasses include APH(4)-I, APH(6)-I, APH(9)-I, APH(3′)-I through IX, APH(3′)-XV, APH(2″)-I through IV, APH(3″)-I and APH(7″)-I (Alcock et al., 2023). The APH(3′)-I subclass, showing resistance to lividomycin, ribostamycin, kanamycin, paromomycin, and neomycin, is composed of three enzymes, and the resistance genes encoding them are generally related to MGEs and are encoded in wide-host-range plasmids (Vakulenko and Mobashery, 2003; Ramirez and Tolmasky, 2010).

In this study, a novel chromosome-encoded aminoglycoside 3′-*O*-phosphotransferase gene designated *aph(3′)-Id* was identified, and its molecular characteristics were characterized through whole-genome sequencing and bioinformatic and kinetic analyses.

## Materials and methods

### Bacteria and whole-genome sequencing

The bacterium DW18 was isolated from the sewage of an animal farm in Wenzhou, China. The collection and isolation methods for DW18 were as follows: a sterile cotton swab was dipped into sewage in the farm sewer line and then immediately placed into a sterile screw-cap specimen collection tube with saline solution. The isolate was streaked on Luria–Bertani agar plates to obtain single colonies, some of which were randomly selected and then preserved in LB broth with 25% glycerol at −80°C. DNA sequencing of DW18 was performed on the Illumina NovaSeq and PacBio RS II platforms (Shanghai Personal Biotechnology Co., Ltd., Shanghai, China). The Illumina short reads were assembled by SKESA v2.4.0 (Souvorov et al., 2018). Unicycler v0.4.8 was used for hybrid assembly of the PacBio long reads and confirming the cyclization of the whole-genome assembly (Wick et al., 2017). Pilon improved the quality of genomic assembly sketches by mapping Illumina short reads onto the assembly to correct possible incorrect assembly (Walker et al., 2014). Species identification was carried out first by 16S rRNA gene homology and then whole-genome average nucleotide identity (ANI) analyses (Konstantinidis and Tiedje, 2005; Richter and Rosselló-Móra, 2009). The calculation of ANI was performed by FastANI (Jain et al., 2018). The bacteria and plasmids used in this study are shown in Table 1.

**Table 1:** Bacteria and plasmids used in this work.

Strain or plasmid	Characteristic(s)	Reference
DW18	The wild-type strain of *Kluyvera intermedia* DW18	This study
DH5α	*E. coli* DH5α was used to clone the *aph(3′)-Id* gene	Laboratory collection
BL21	*E. coli* BL21 was used as a host to express the *aph(3′)-Id* gene	Laboratory collection
ATCC25922	*E. coli* ATCC25922 was used as a quality control for antimicrobial susceptibility testing	Laboratory collection
pMD19/DH5α	DH5α carrying the plasmid pMD19-T Simple Vector	Laboratory collection
pMD19-*aph(3′)-Id*/DH5α	DH5α carrying the recombinant plasmid pMD19-*aph(3′)-Id*	This study
pCold I-*aph(3′)-Id*/BL21	BL21 carrying the recombinant plasmid pCold I-*aph(3′)-Id*	This study

### Antimicrobial susceptibility testing

The agar dilution method was used to determine the minimum inhibitory concentrations (MICs) of the antimicrobials. Susceptibility patterns were interpreted on the basis of the Clinical and Laboratory Standards Institute guidelines M100 (32nd Edition, 2022) for Enterobacteriaceae. The reference strain used for quality control was *E. coli* ATCC 25922. All 16 antimicrobials used in this work are shown in Table 2.

**Table 2:** MICs of various antimicrobials for five bacterial strains (μg/mL).

Class	Antimicrobial	DW18	pMD19-*aph(3′)-Id*/DH5α	pMD19/DH5α	DH5α	ATCC 25922
Aminoglycosides	Spectinomycin	16	8	8	8	8
	Streptomycin	4	2	2	2	4
	Sisomicin	0.25	0.25	0.25	0.25	0.25
	Ribostamycin	64	1,024	2	2	4
	Tobramycin	0.5	0.5	0.5	0.25	0.5
	Gentamicin	0.25	0.25	0.25	0.25	0.5
	Kanamycin	32	1,024	1	1	2
	Neomycin	2	16	1	1	1
	Paromomycin	32	256	2	2	4
	Amikacin	1	1	1	1	2
β-Lactams	Penicillin	2,048	/	/	/	32
	Ampicillin	256	/	/	/	8
	Cefoxitin	512	/	/	/	2
	Cefazolin	64	/	/	/	2
	Ceftazidime	1	/	/	/	0.25
	Aztreonam	0.25	/	/	/	0.25

/, the MICs of the antimicrobials for the corresponding strains were not tested.

### Cloning of the *aph(3′)-Id* gene

The promoter region and coding sequence of the novel resistance gene *aph(3′)-Id* were amplified by polymerase chain reaction (PCR). The primers and annealing temperatures used for PCR are listed in Table 3. The PCR products were inserted into T-Vector pMD19 (Simple) (Takara Bio, Inc., Dalian, China) using a T4 DNA ligase cloning kit (Takara Bio, Inc., Dalian, China). The recombinant plasmid was transformed into competent cells of *E. coli* DH5α by the calcium chloride method, and then LB broth was added, and the cells were resuscitated by shaking at 37°C. The transformants were screened on LB solid culture medium with 100 μg/mL ampicillin. For the expression of the APH(3′)-Id protein, the ORF of *aph(3′)-Id* was PCR-amplified using forward and reverse primers with *Bam*HI and *Hind*III restriction sites, respectively, at the 5′-ends. The PCR product was digested with the restriction endonucleases *Hind*III and *Bam*HI and then cloned into the pCold I vector treated with the same restriction endonucleases. The recombinant plasmid (pCold I-*aph(3′)-Id*) was transformed into competent *E. coli* BL21 cells by the calcium chloride method. The transformants were screened on LB agar plates with 100 μg/mL ampicillin. The cloned insert sequences of the recombinant plasmids were confirmed by PCR and Sanger sequencing.

**Table 3:** Primers for cloning the *aph(3′)-Id* gene.

Primers^a^	Sequence (5′ → 3′)	Restriction endonuclease site	Vector	Annealing temperature (°C)	Amplicon size (bp)
pro- *aph(3′)-Id*-F	AGACAGTGTAAGTGCTTACAGTACGAAC		T-Vector pMD19	60	945
pro- *aph(3′)-Id*-R	GATTTGCCATTAAGTTGCAGTGATCCC		T-Vector pMD19	60	945
orf*- aph(3′)-Id*-F	CGGGATCCCTGGTGCCGCGCGGCAGCATGAATCATAGTCAAAGAGAAACATCGTGC	*Bam*HI + Thrombin	pCold I	59	868
orf*- aph(3′)-Id*-R	CCAAGCTTCTGGTGCCGCGCGGCAGCTTAGAAAAATTCATCAAGCATTAAATGAAACTGCAAC	*Hind*III	pCold I	59	868

a Primers starting with “pro” were used to clone the ORF with the promoter region of the *aph(3′)-Id* gene; primers starting with “orf” were used to clone the ORF of the *aph(3′)-Id* gene.

### Expression and purification of the APH(3′)-Id enzyme

The recombinant strain (pCold I-*aph(3′)-Id*/BL21) for the expression of APH(3′)-Id was cultured in 3 mL of LB broth with 100 μg/mL ampicillin at 37°C for 16 h. Then, 1 mL of the bacterial culture was added to 100 mL of LB broth for continuous culture until the OD_600_ value reached 0.6–0.8 (Qing et al., 2004). After refrigeration for half an hour, a final concentration of 1 mM IPTG was added to induce the expression of APH(3′)-Id. Cultivation was continued for 16 h at 18°C, and the culture was centrifuged to collect cells. The cells were resuspended in 4 mL of nondenatured lysate (50 mM NaH_2_PO_4_, 300 mM NaCl) and disrupted by ultrasonication. The recombinant protein in the supernatant collected after centrifugation at 8,000 × g for 30 min was purified using His-tag Purification Resin and eluted by the nondenatured eluent (300 mM NaCl, 50 mM imidazole, 50 mM NaH_2_PO_4_) according to the instructions of the His-tag Protein Purification Kit (Reductant & Chelator-resistant) (Beyotime, Shanghai, China). The His tag of the recombinant protein was removed by thrombin with incubation at 20°C for 16 h. The purity of APH(3′)-Id was confirmed by SDS–PAGE, and the concentration was spectrophotometrically examined at 562 nm by the standard curve established using a BCA protein assay kit (Beyotime, Shanghai, China). The protein was stored in dissolution buffer (0.5 M NaCl, 20 mM Tris–HCl; pH 8.0) and preserved at −80°C for subsequent enzyme kinetics analysis.

### Enzyme kinetics determination

The kinetic parameters of APH(3′)-Id were determined as described in previous reports (Toth et al., 2013; Lu et al., 2021) with slight modifications. The phosphorylation of aminoglycosides was coupled to NADH oxidation by pyruvate kinase (PK) and lactate dehydrogenase (LDH). The reduction of NADH at 340 nm was monitored by a SpectraMax M5 multifunctional microplate reader (Molecular Devices, United States) to reflect the speed of aminoglycoside phosphorylation. The reaction mixture contained 1 mM phosphoenolpyruvate, 1 mM MgCl_2_, 100 mM HEPES (pH 7.5), 600 μM NADH, 2 mM KCl, a commercial mixture of PK and LDH (Sigma P0294; 18–26 U/mL PK and 25–35 U/mL LDH in final concentration), 1 mM ATP, 30–40 nM APH(3′)-Id and aminoglycosides (5–400 μM) in a total volume of 250 μL. The steady-state velocities were calculated from the linear period of the reaction process curve and applied to plot the substrate concentration-velocity curve. The data were fit by nonlinear regression with the velocity as a function of substrate using the Michaelis–Menten equation. Curve fitting and calculation of the dynamic parameters *K*_m_ and *k*_cat_ were completed in GraphPad Prism 8.0.2 (GraphPad Software, Inc.).

### Bioinformatics analysis

Prokka v1.14.6 (Seemann, 2014) was used to predict the potential open reading frames (ORFs) of the assembled genome sequence, and the deduced proteins were annotated using DIAMOND v2.0.11 (Buchfink et al., 2021) according to the Comprehensive Antibiotic Resistance Database (CARD) (McArthur et al., 2013) to identify antimicrobial resistance genes. The annotation parameters were set as follows: E-value <1E-9, identity >95% and coverage >85%. Multiple sequence alignment and visualization were conducted using MAFFT v7.490 (Katoh and Standley, 2013) and GeneDoc v2.7 (Nicholas, 1997). Construction of phylogenetic trees and their visualization were conducted using IQ-TREE v1.6.9 (Nguyen et al., 2015) and iTOL v6.7 (Letunic and Bork, 2021). The crystal structure of the aminoglycoside phosphotransferase APH(3′)-Ia (SMTL ID: 3r78.1), which shared the highest amino acid identity of 77.49% with APH(3′)-Id, was used as a template for homology modeling of APH(3′)-Id in SWISS-MODEL (Waterhouse et al., 2018) to predict the tertiary structure. The tertiary structure alignment of the APH(3′)-Id model and kanamycin-bound APH(3′)-Ia (PDB 4FEU chain B) was performed by PyMOL (Schrödinger, 2023). A map of the genetic environment surrounding *aph(3′)-Id* was generated by clinker v0.0.24 (Gilchrist and Chooi, 2021). The Expasy ProtParam Tool (Gasteiger et al., 2003) was used for prediction of the molecular weight and pI value of the protein APH(3′)-Id.

### Nucleotide sequence accession numbers

The following accession numbers have been assigned for the complete genome sequence of *Kluyvera intermedia* DW18 in GenBank: CP123488 for chromosome, CP123489 for plasmid pDW18-1 and CP123490 for plasmid pDW18-2. The nucleotide sequence of the novel AME gene *aph(3′)-Id* has been assigned GenBank accession number OQ819314.

## Results and discussion

### Identification and characteristics of *Kluyvera intermedia* DW18

The 16S rRNA gene of DW18 shared the highest similarity of 100% (100% identity and 100% coverage) with that of *K. intermedia* MAG 4195 (GCA_029202345.1). The genome sequence of DW18 shared 98.95% ANI with that of *K. intermedia* N2-1 (GCA_009649915.1), which exceeded the threshold of 95% to define a bacterial species. Currently, the ANI between genomes is considered the best alternative to DDH and 16S rRNA gene homology analyses as the gold standard for prokaryotic classification, and the ANI threshold for species membership was empirically defined as 95% (Konstantinidis and Tiedje, 2005; Richter and Rosselló-Móra, 2009). Therefore, the isolate DW18 in this study was classified into the species *K. intermedia* and designated *K. intermedia* DW18.

### General features of the *Kluyvera intermedia* DW18 genome

The complete genome of *K. intermedia* DW18 was assembled, and it was found to consist of a chromosome and two circular plasmids. The chromosome is approximately 4.62 Mb in length and encodes 4,417 ORFs with an average GC content of 52.79%. The two circular plasmids were designated pDW18-1 and pDW18-2 (Table 4). pDW18-1 is 4,455 bp in length and encodes 9 ORFs, while pDW18-2 is 3,612 bp in length and encodes 6 ORFs. In the NCBI database, a total of 23 *K. intermedia* genomes were present, and only four (*K. intermedia* N2-1, GCA_009649915.1, *K. intermedia* HR2, GCA_009650415.1, *K. intermedia* NCTC12125, GCA_900635475.1, and *K. intermedia* MAG 4159, GCA_029202345.1) of them had complete genome sequences. Among the four strains with complete genome sequences, two (*K. intermedia* N2-1 and *K. intermedia* HR2) carried a large plasmid each (pN2-1, 245,785 bp in length, and pHR2-1, 245,785 bp in length). The other strains, NCTC12125 and MAG 4159, did not carry any plasmid. DW18, however, carried two small plasmids, pDW18-1 (4,455 bp) and pDW18-2 (3,612 bp). The chromosome size of the five *K. intermedia* strains with complete genomes (including DW18 in this work) varied slightly, ranging from 4.62 Mb (DW18) to 4.74 Mb (NCTC12125).

**Table 4:** General features of the *Kluyvera intermedia* DW18 genome.

	Chromosome	pDW18-1	pDW18-2
Size (bp)	4,624,787	4,455	3,612
GC content (%)	52.79	52.44	45.62
Predicted coding sequences (CDSs)	4,417	9	6
Known proteins	3,813	2	0
Hypothetical proteins	604	7	6
Protein coding (%)	79.02	100	85.71
Average ORF length (bp)	917.3	408.3	240.1
Average protein length (aa)	301.7	135.2	88.3
tRNAs	86	/	1
rRNA operons	(16S-23S-5S)×7 (16S-23S-5S-5S)×1	/	/

### Resistance profiles of *Kluyvera intermedia* DW18

Of the 16 antimicrobials from two classes (aminoglycosides and β-lactams) tested, DW18 showed higher MIC levels for eight, including four aminoglycosides (ribostamycin 64 μg/mL, kanamycin 32 μg/mL, paromomycin 32 μg/mL, and spectinomycin 16 μg/mL) and four β-lactams (penicillin 2048 μg/mL, cefoxitin 512 μg/mL, ampicillin 256 μg/mL, and cefazolin 64 μg/mL) (Table 2). On the one hand, for β-lactams, the resistance profile of *K. intermedia* DW18 was generally consistent with previous reports, but on the other hand, for aminoglycosides, it showed different resistance phenotypes. Previous studies showed that *K. intermedia* was resistant to a variety of β-lactam antibiotics, such as penicillin, amoxycillin, and cefoxitin, but was susceptible to aminoglycosides (Stock, 2002). Some isolates also showed resistance to carbapenems and other cephalosporins such as imipenem, meropenem, cefepime, and ceftriaxone (Ribeiro et al., 2014; Paskova et al., 2018).

In our present project studying the resistance status of animal bacteria, we sequenced the draft genomes of several strains isolated from animal specimens. When analyzing the resistance genotype of the DW18 genome based on the CARD with thresholds of identity >95% and coverage >85%, we found that no functionally characterized aminoglycoside and β-lactam resistance gene was annotated in the genome even though it showed high MIC levels for some aminoglycosides and β-lactams.

To determine the potential resistance mechanism of DW18 against aminoglycosides, we examined the annotation data of the genome sequence and found that among the predicted genes, the gene showing the highest identity with the functionally characterized aminoglycoside resistance gene was an *aph(3′)-Ia*-like gene (finally designated *aph(3′)-Id* in this work), which showed amino acid similarity (100% coverage and 77.49% identity) with APH(3′)-Ia (CAE51638.1). To determine whether the predicted resistance gene was indeed functional, we cloned the ORF of the gene with its promoter region into the T-vector pMD19, and the resistance function of this gene was confirmed. Compared with the control strains (DH5α or DH5α carrying pMD19), the recombinant strain (pMD19-*aph(3′)-Id*/DH5α) showed 1,024-, 512-, 128-, and 16-fold increased MICs for kanamycin, ribostamycin, paromomycin and neomycin, respectively. No significant increases in the MICs for the other 6 aminoglycosides tested were observed (Table 2). There are only three other *aph(3′)-I* genes [*aph(3′)-Ia*, *aph(3′)-Ib* and *aphA15*] present in CARD, and only *aph(3′)-Ia* and *aphA15* had the resistance profile documented (Siregar et al., 1995; Riccio et al., 2001). *aph(3′)-Id*, *aph(3′)-Ia* and *aphA15* all showed higher MIC values for kanamycin and neomycin. The *aph(3′)-Id* gene in this work did not confer resistance to gentamicin or amikacin; however, the *aph(3′)-Ia* gene showed high MICs for gentamicin (>1,024 μg/mL) and amikacin (8 μg/mL). According to the evolutionary relationship, substrate spectrum and nomenclature proposed for genes encoding AMEs (Shaw et al., 1993), the novel resistance gene in this work was thus designated *aph(3′)-Id.*

### Homologs of the novel aminoglycoside 3’-*O*-phosphotransferase APH(3′)-Id

When analyzing the distribution of *aph(3′)-Id* and *aph(3′)-Id-*like genes, only 4 genes with amino acid identities >80% were found according to the BLAST search against the NCBI nonredundant protein database. These genes were derived from *K. intermedia* (WEJ84261.1, WP_153742217.1, WP_062773153.1) and *Klebsiella* sp. (WP_181482651.1). The deduced amino acid sequence of APH(3′)-Id shared the highest identity of 99.26% with a hypothetical APH(3′)-I family aminoglycoside *O*-phosphotransferase (WEJ84261.1) from *K. intermedia*. The other two proteins from *K. intermedia* also shared high identities of 98.89% (WP_153742217.1) and 97.05% (WP_062773153.1) with APH(3′)-Id. However, the protein from *Klebsiella* sp. shared the lowest identity of 80.07% (WP_181482651.1) with APH(3′)-Id. This indicated that APH(3′)-Id was relatively conserved within the species *K. intermedia.*

**Figure 1:**
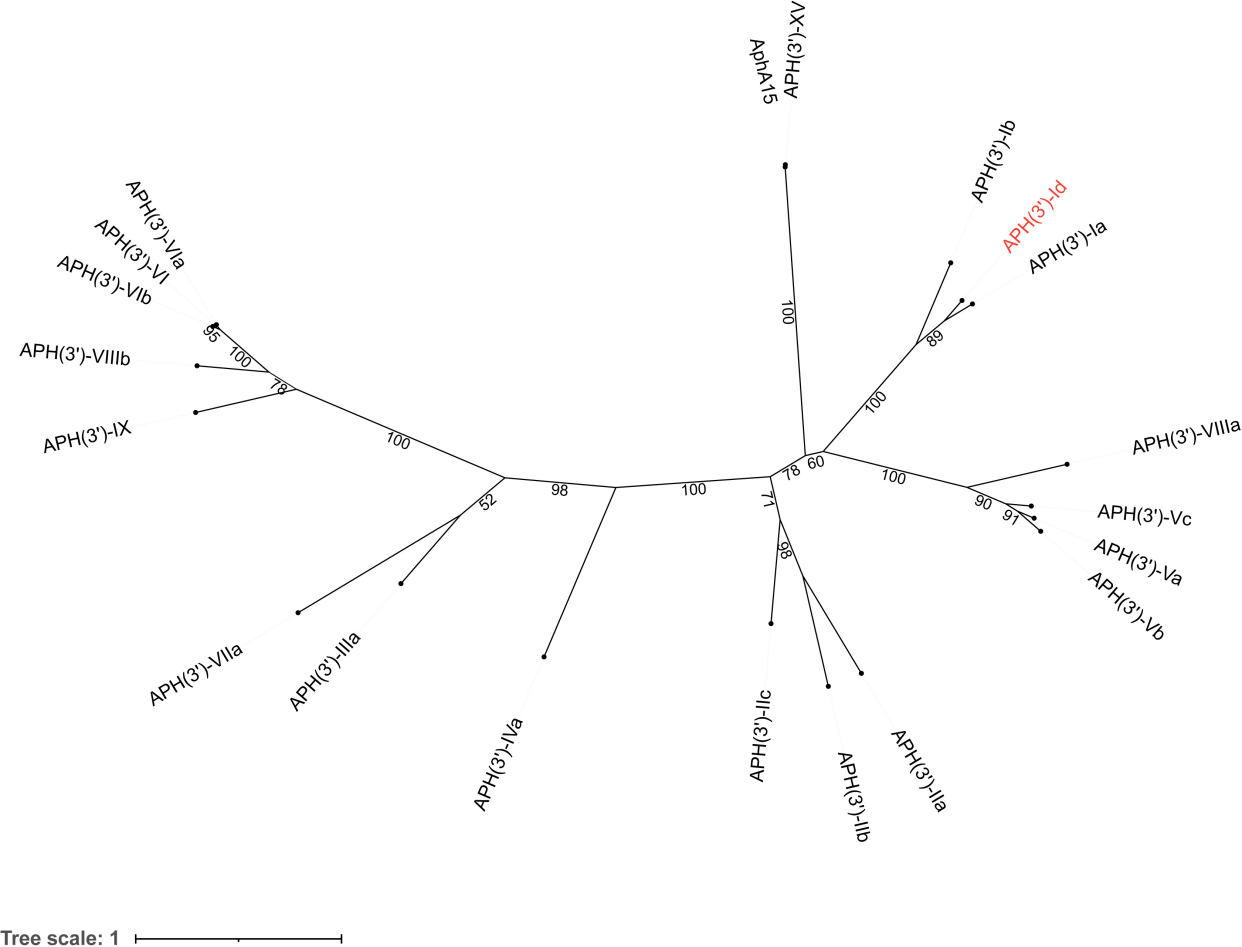
A phylogenetic tree showing the relationship of APH(3′)-Id with other APH(3′) enzymes. APH(3′)-Id is colored in red. The numbers on the branches are bootstrapping values. The proteins and their accession numbers: APH(3′)-Ia (CAE51638.1), APH(3′)-Ib (AAA26412.1), APH(3′)-XV (ABY48974.1), AphA15 (CAD91341.1), APH(3′)-VIIIa (AAG11411.2), APH(3′)-Vc (AAB21326.1), APH(3′)-Va (AAA26699.1), APH(3′)-Vb (AAC32025.1), APH(3′)-IIa (CAA23892.1), APH(3′)-IIb (CAA62365.1′), APH(3′)-IIc (ADQ43421.1), APH(3′)-IVa (CAA27061.1), APH(3′)-IIIa (AGV10830.1), APH(3′)-VIIa (AAA76822.1), APH(3′)-IX (ENV34035.1), APH(3′)-VIIIb (EPF73263.1), APH(3′)-VIb (CAF29483.1), APH(3′)-VI (AGI04227.1), and APH(3′)-VIa (CAA30578.1).

The novel aminoglycoside 3′-*O*-phosphotransferase gene *aph(3′)-Id* was 816 bp in length and encoded a protein of 271 amino acids (30.69 kDa) with a pI value of 4.90. APH(3′)-Id shared the highest amino acid identities of 77.49, 58.67 and 39.85% with the functionally characterized APH(3′) proteins APH(3′)-Ia (CAE51638.1), APH(3′)-Ib (AAA26412.1) and APH(3′)-IIc (ADQ43421.1), respectively. Furthermore, a phylogenetic tree that included all 19 functionally characterized APH(3′) enzymes collected from the CARD database showed that APH(3′)-Id clustered closest to APH(3′)-I enzymes (Figure 1). Currently, the *aph(3′)-Id* genes were mainly found in the chromosomes of *Kluyvera intermedia*. The *aph(3′)-Ib* and *aph(3′)-IIc* genes were only found in *Pseudomonas aeruginosa* and *Stenotrophomonas maltophilia*, respectively. The *aph(3′)-Ia* gene, however, was initially identified in the translocator element Tn*903* of *Escherichia coli* (Oka et al., 1981), but now is widely distributed in a variety of bacterial genera, such as *Acinetobacter*, *Aeromonas*, *Avibacterium*, *Brucella*, and *Enterobacter*. This suggested that *aph(3′)-Id* may not spread as rapidly and widely across multiple strains as *aph(3′)-Ia*.

**Figure 2:**
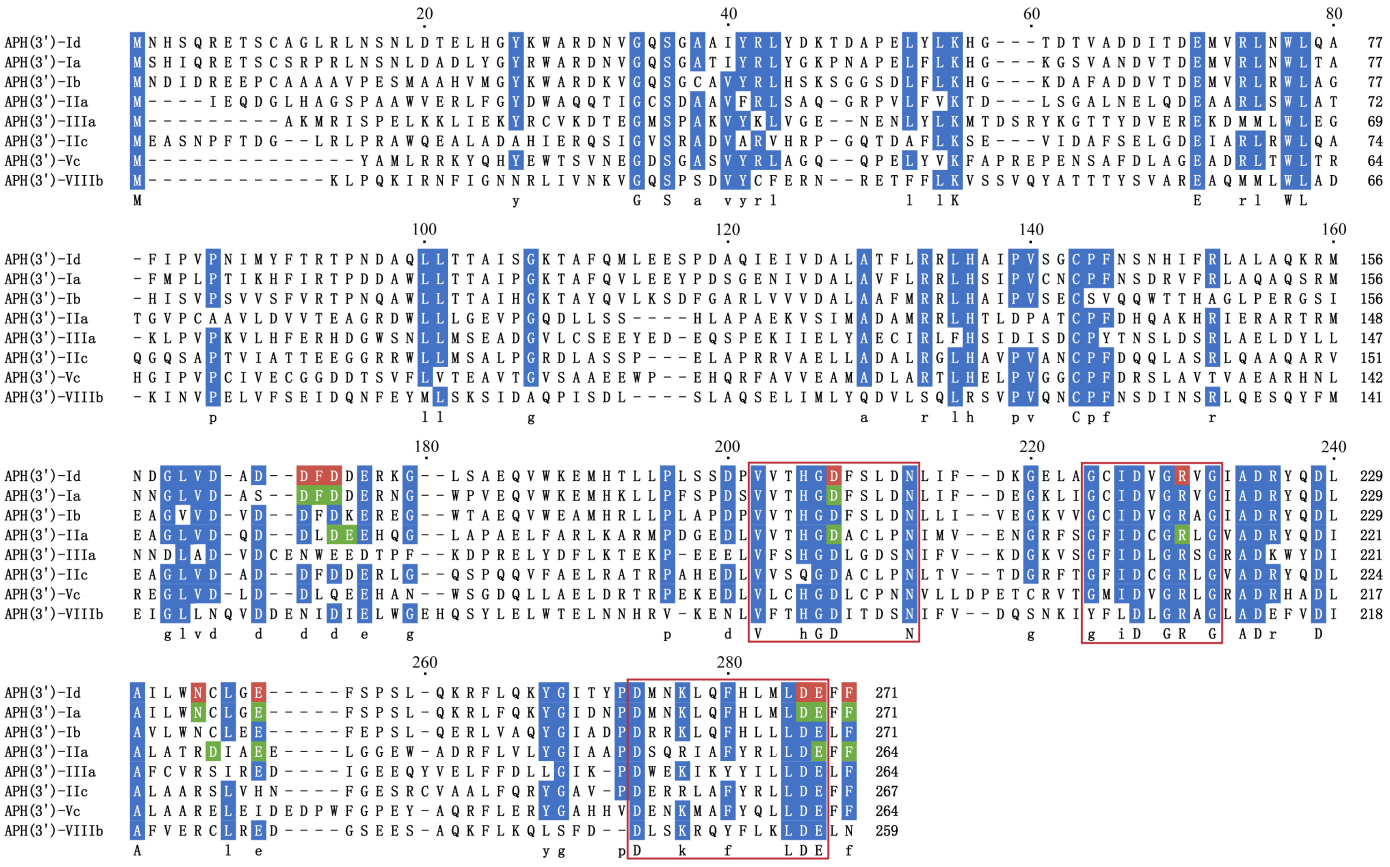
Multiple sequence alignment of APH(3′)-Id with other functionally characterized relatives. Dark blue indicates highly similar residues; red indicates residues related to kanamycin binding sites in APH(3′)-Id; green indicates residues representing kanamycin binding sites in APH(3′)-Ia and APH(3′)-IIa; three conserved functional domains of the aminoglycoside phosphotransferase family are framed in red squares; gaps are represented using hyphens. The numbers on the right represent the length of the corresponding sequence. Accession numbers of APH(3′) proteins: APH(3′)-Ia (CAE51638.1), APH(3′)-Ib (AAA26412.1), APH(3′)-IIa (CAA23892.1), APH(3′)-IIIa (AGV10830.1), APH(3′)-IIc (ADQ43421.1), APH(3′)-Vc (AAB21326.1) and APH(3′)-VIIIb (EPF73263.1).

**Figure 3:**
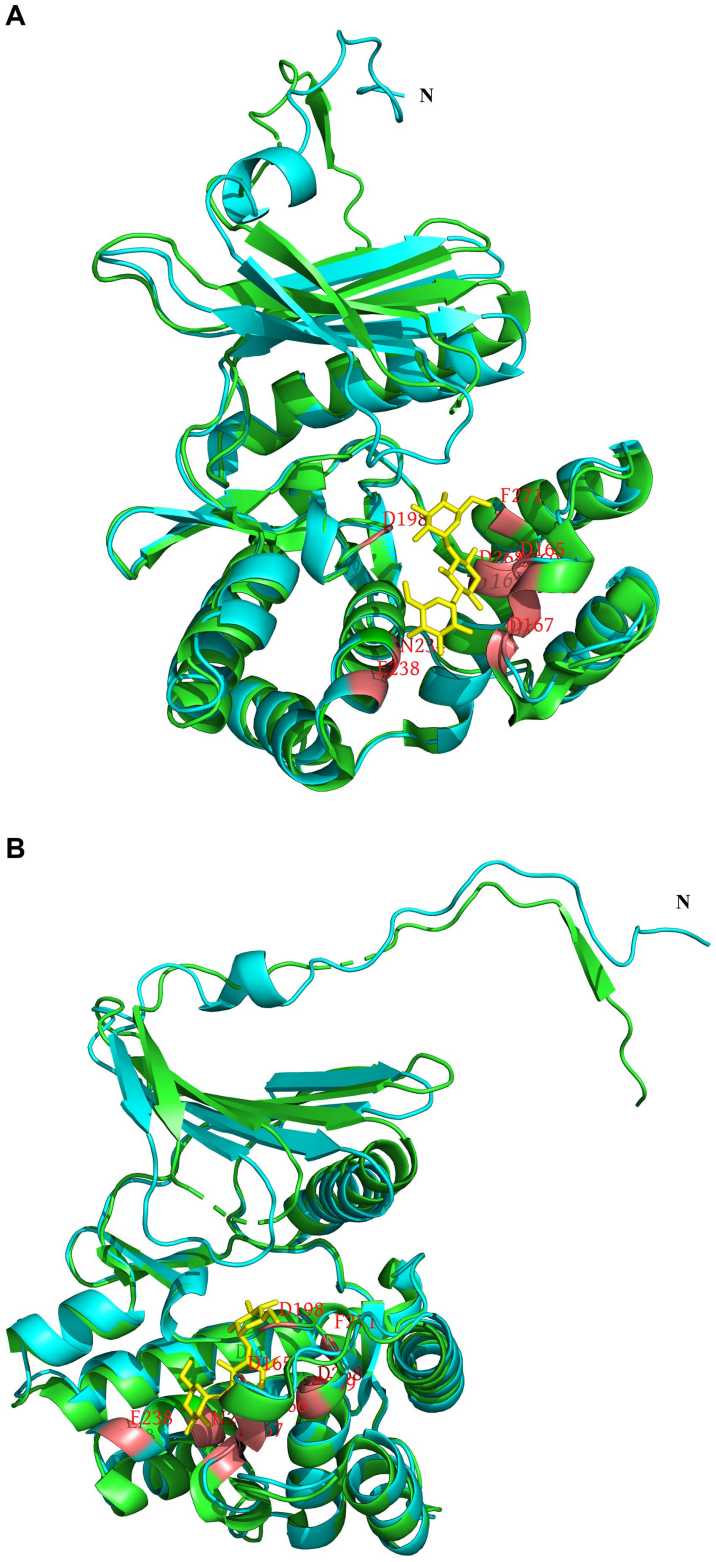
Tertiary structure alignment of the APH(3′)-Id model and kanamycin-bound APH(3′)-Ia. **(A,B)** are different illustrations of the same alignment. The APH(3′)-Id model is colored blue; APH(3′)-Ia is colored green; the position of amino acid residues bound with kanamycin is colored red in the APH(3′)-Id model and APH(3′)-Ia; kanamycin is colored yellow.

To analyze the structure related to the resistance function of APH(3′)-Id, multiple sequence alignment of APH(3′)-Id and other functionally characterized APH(3′) proteins was performed (Figure 2). The results showed that APH(3′)-Id contained three conserved functional domains of the aminoglycoside phosphotransferase family, including Motif 1 (V--HGD----N), Motif 2 (G--D-GR-G) and Motif 3 (D--R/K--F/Y---LDE) (Shaw et al., 1993) (Figure 2). Six of eight active residues responsible for the phosphorylation of kanamycin in APH(3′)-IIa were identified in APH(3′)-Id (D167, D198, R219, E238, E269 and F271) (Nurizzo et al., 2003) (Figure 2). All nine kanamycin binding sites of APH(3′)-Ia were also identified in APH(3′)-Id (D165, F166, D167, D198, N234, E238, D268, E269, and F271) (Stogios et al., 2013) (Figures 2, 3). The tertiary structure alignment of the APH(3′)-Id model and kanamycin-bound APH(3′)-Ia showed that the overall structures of the two were similar, especially the kanamycin binding position. Similar to APH(3′)-Ia, the ATP binding sites of APH(3′)-Id, located in the hinge region between the N- and the C-terminal lobe of the bilobal fold, also had a similar spatial structure to that of eukaryotic protein kinases. The difference was that APH(3′)-Ia had a β-sheet (R6, E7, T8, C9), while APH(3′)-Id had an α-helix (L19, D20, T21, E22, L23), at the N terminus (Figure 3).

### Kinetic parameters of APH(3′)-Id

The induced expressed protein APH(3′)-Id was soluble (Supplementary Figure S1). After nickel column purification, His-tag removal and ultrafiltration concentration, 2 mL of 1.01 mg/mL (33.06 μM) APH(3′)-Id protein was obtained (Supplementary Figures S2, S3). The kinetic analysis of the phosphotransferase APH(3′)-Id showed that the purified APH(3′)-Id could phosphorylate four substrates, including ribostamycin, kanamycin, neomycin and paromomycin, which was consistent with the results for APH(3′)-I enzymes reported previously (Ramirez and Tolmasky, 2010). In the APH(3′)-I subclass, the enzyme with the closest evolutionary relationship to APH(3′)-Id was APH(3′)-Ia. By comparing the kinetic parameters between APH(3′)-Id and APH(3′)-Ia (Siregar et al., 1995), it was found that the affinity and catalytic efficiencies of APH(3′)-Id for the substrates were slightly weaker than those of APH(3′)-Ia. For example, when kanamycin was the substrate, the *K*_m_ of APH(3′)-Ia was lower than that of APH(3′)-Id (1.2 ± 0.2 μM vs. 10.04 ± 2.95 μM), while the *k*_cat_*/K*_m_ of APH(3′)-Ia was higher than that of APH(3′)-Id (8.5 × 10^7^ M^−1^/s^−1^ vs. 1.10 × 10^5^ M^−1^/s^−1^). A similar result was observed when neomycin was the substrate. The *K*_m_ of APH(3′)-Ia for neomycin was lower than that of APH(3′)-Id (3.6 ± 0.3 μM vs. 18.70 ± 1.51 μM), while the *k*_cat_*/K*_m_ of APH(3′)-Ia for neomycin was higher than that of APH(3′)-Id (5.1 × 10^7^ M^−1^/s^−1^ vs. 1.73 × 10^5^ M^−1^/s^−1^).

The substrate spectrum of APH(3′)-Id was also consistent with the resistance profile shown by the recombinant strain (pMD19-*aph(3′)-Id*/DH5α). Consistent with the MIC results, no phosphotransferase activity was found for gentamicin or streptomycin (Table 5). The catalytic efficiencies of APH(3′)-Id varied from the MIC values of the cloned *aph(3′)-Id.* APH(3′)-Id showed the highest catalytic efficiency for ribostamycin [*k*_cat_*/K*_m_ of (4.96 ± 1.63) × 10^5^ M^−1^/s^−1^], followed by paromomycin [*k*_cat_*/K*_m_ of (2.18 ± 0.21) × 10^5^ M^−1^/s^−1^], neomycin [*k*_cat_*/K*_m_ of (1.73 ± 0.20) × 10^5^ M^−1^/s^−1^], and kanamycin [*k*_cat_*/K*_m_ of (1.10 ± 0.18) × 10^5^ M^−1^/s^−1^]. However, the cloned *aph(3′)-Id* showed the highest MIC increase for kanamycin (1024-fold), followed by ribostamycin (512-fold), paromomycin (128-fold) and neomycin (16-fold). Neomycin had a high enzymatic catalytic efficiency but a low MIC value. It is possible that it was poorly transferred into the cell and could not reach the saturation concentration of the enzyme, so the enzyme could not fully perform its catalytic function (Siregar et al., 1995). In addition, APH(3′)-Id showed a high MIC level for kanamycin, while the catalytic efficiency was not very high. Different phosphate donors may result in different catalytic efficiencies of enzymes. ATP was used as the phosphate donor in this study. In a previous kinetic assay, the *k*_cat_ of kanamycin phosphorylated by APH(3′)-Ia with GTP as a donor was 10-fold higher than that with ATP as the donor (Stogios et al., 2013).

**Table 5:** Steady-state kinetic parameters for APH(3′)-Id.

Substrate	*K*_m_ (μM)^a^	*k*_cat_ (s^−1^)	*k*_cat_*/K*_m_ (M^−1^/s^−1^)
Neomycin	18.70 ± 1.51	3.221 ± 0.102	(1.73 ± 0.20) × 10^5^
Kanamycin	10.04 ± 2.95	1.056 ± 0.114	(1.10 ± 0.18) × 10^5^
Ribostamycin	3.831 ± 1.57	1.730 ± 0.023	(4.96 ± 1.63) × 10^5^
Paromomycin	12.19 ± 1.56	2.637 ± 0.072	(2.18 ± 0.21) × 10^5^
Gentamicin	NA^b^	NA	NA
Streptomycin	NA	NA	NA

a Values are the means ± standard deviations.

b NA, no phosphotransferase activity was detected.

As mentioned above, the substrate spectrum of APH(3′)-Id appeared to be similar to that of the other APH(3′)-I enzymes, which showed modification activity against several aminoglycosides, including kanamycin, ribostamycin, neomycin, paromomycin, lividomycin, gentamicin, butirosin and amikacin (Shaw et al., 1993; Siregar et al., 1995; Ramirez and Tolmasky, 2010). The substrate spectrum of the APH(3′)-II enzymes was the same with APH(3′)-I subclass (Shaw et al., 1993; Lu et al., 2021). APH(3′)-III enzymes had a broader substrate spectrum than APH(3′)-II, showing modification activity for isepamicin (Shaw et al., 1993; Ramirez and Tolmasky, 2010). In general, the substrate profile of APH(3′)-Id was consistent with that of the APH(3′)-I enzymes, and it further supported that this novel enzyme can be concluded into the APH(3′)-I subclass.

### Genetic environment and distribution of *aph(3′)-Id*

**Figure 4:**
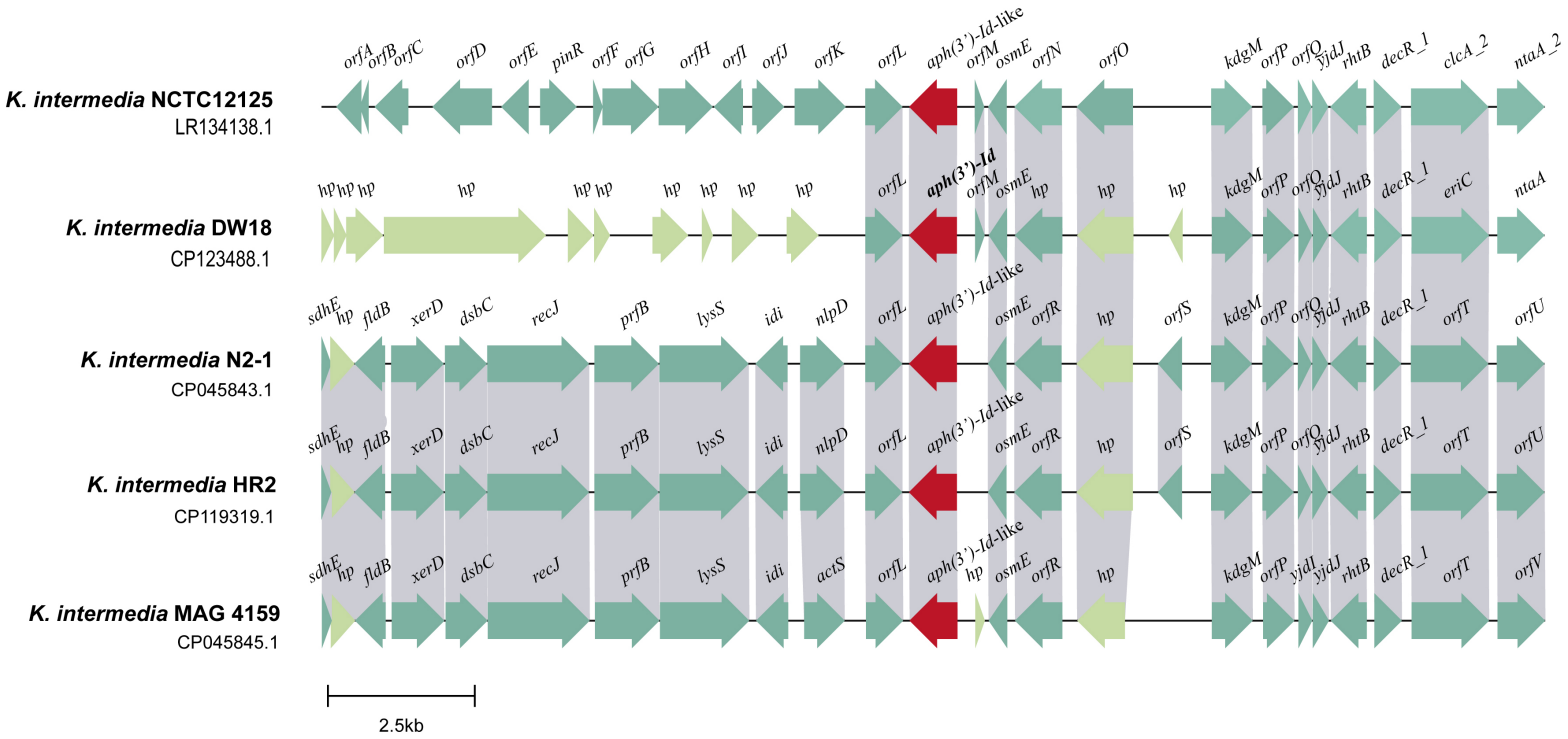
Genetic environment of the *aph(3′)-Id* and *aph(3′)-Id*-like genes. ORFs are shown as arrows drawn to scale to indicate the direction of transcription and colored based on gene function classification. Regions with ≥80% nucleotide identity are colored gray. hp: hypothetical protein.

The *aph(3′)-Id* gene is located in the chromosome of *K. intermedia* DW18. To explore its genetic context, the structure of an approximately 20 kb sequence with *aph(3′)-Id* at the center was analyzed (Figure 4). When using the nucleotide sequence of *aph(3′)-Id* as a query to search the NCBI nonredundant nucleotide database, a total of six *aph(3′)-Id*-like genes with identities of more than 80.0% were found. Of these six *aph(3′)-Id*-like genes, four were from the same species, *K. intermedia,* and each shared particularly high similarity (100% coverage and > 98% identity) with *aph(3′)-Id*, while the other two *aph(3′)-Id*-like genes were from *Klebsiella* sp. (CP056483.1 and CP055481.1) and shared a lower similarity (both with 100% coverage and 81.13% identity) with *aph(3′)-Id*. The genetic context of *aph(3′)-Id* with the four *aph(3′)-Id*-like genes that shared higher identities with *aph(3′)-Id* was then analyzed.

Multisequence alignment of the five 20 kb sequences from *K. intermedia* (including one from this work) demonstrated that the downstream regions of the *aph(3′)-Id*(−like) genes were similar among them. This region of DW18 is mainly composed of genes related to transcription (*osmE* and *decR_1*) and transport (*eriC*, *rhtB* and *kdgM*). Nitrilotriacetate is an industrial chelating agent extensively used in textile and wastewater treatment processes. The product of the *ntaA* gene, nitrilotriacetate monooxygenase component A, is the core component of the nitrilotriacetate biodegradation process and may help prevent the harm caused by chelating agents in sewage (Kim K. J. et al., 2006). However, the upstream fragments were completely different between the gene from this work and any of the other four, except the *orfL* gene next to the *aph(3′)-Id*(−like) genes. The product of the *orfL* gene belongs to the multifunctional glutathionine S-transferase family, members of which can detoxify several chemical pollutants in the environment, such as 2,4,6-trinitrotoluen (TNT), toxic chlorinated organic compounds and anthracene (Mcguinness et al., 2007; Dixit et al., 2011; Gunning et al., 2014). No MGE was found in the flanking regions of the *aph(3′)-Id*(−like) genes (Figure 4). These results suggest that *aph(3′)-Id* may be an intrinsic gene of *Kluyvera*, and modification of aminoglycosides may be one of its normal physiological functions to protect the organism from environmental drugs. Although no MGE was detected near *aph(3′)-Id*, the possibility of horizontal transfer of this resistance gene between bacteria of different species or genera in the wild in the long run cannot be ruled out.

## Conclusion

In this study, we reported the complete genome sequence of *K. intermedia* DW18 isolated from sewage in an animal farm and identified and characterized a novel aminoglycoside phosphotransferase gene, designated *aph(3′)-Id*, encoded in its chromosome. APH(3′)-Id shared the highest amino acid identity of 77.49% with the aminoglycoside 3′-*O*-phosphotransferase APH(3′)-Ia. APH(3′)-Id, consistent with the other enzymes of the APH(3′)-I subclass, conferred strong modification activity for specific aminoglycosides. These findings could help clinicians choose effective antimicrobials to treat infections caused by this bacterial species.

## Data availability statement

The datasets presented in this study can be found in online repositories. The names of the repository/repositories and accession number(s) can be found in the article/Supplementary material.

## Author contributions

HZ, KL, QB, and DL: conceived and designed the experiments. YS, NL, GZ, YZ, JZ, JL, and TZ: performed the experiments. YS, XZ, QL, and XL: data analysis and interpretation. YS, QB, and DL: drafting of the manuscript. All authors contributed to the article and approved the submitted version.

## Funding

This study was supported by the Science and Technology Project of Wenzhou City, China (N20210001), the Science and Technology Project of Jinhua City, China (2022–2-013, 2022–4-017) and the Zhejiang Provincial Natural Science Foundation of China (LY19C060002 and LQ17H190001).

## Conflict of interest

The authors declare that the research was conducted in the absence of any commercial or financial relationships that could be construed as a potential conflict of interest.

## Publisher’s note

All claims expressed in this article are solely those of the authors and do not necessarily represent those of their affiliated organizations, or those of the publisher, the editors and the reviewers. Any product that may be evaluated in this article, or claim that may be made by its manufacturer, is not guaranteed or endorsed by the publisher.
