# Supplementary material for: Identification and characterization of a novel chromosomal aminoglycoside 3’-O-phosphotransferase, APH(3′)-Id, from Kluyvera intermedia DW18 isolated from the sewage of an animal farm
Source: Front Microbiol. 2023 Aug 28;14:1224464. doi: 10.3389/fmicb.2023.1224464 (PMC10493288; doi:10.3389/fmicb.2023.1224464)
Supplement: Supplementary file 1 [file Presentation_1.pdf]

*pinR*: putative DNA-invertase from lambdoid prophage Rac;

*osmE*: DNA-binding transcriptional activator OsmE;

*kdgM*: oligogalacturonate-specific porin kdgM precursor;

*yjdJ*: putative acyltransferase with acyl-CoA N-acyltransferase domain;

*rhtB*: homoserine/homoserine lactone efflux protein;

*decR_1*: DNA-binding transcriptional activator DecR;

*clcA_2*: H(+)/Cl(−) exchange transporter ClcA;

*ntaA_2*: nitrilotriacetate monooxygenase component A;

*eriC*: Chloride/fluoride channel protein; *ntaA*: nitrilotriacetate monooxygenase component A;

*sdhE*: FAD assembly factor SdhE; *fldB*: flavodoxin FldB;

*xerD*: site-specific tyrosine recombinase XerD;

*dsbC*: bifunctional protein-disulfide isomerase/oxidoreductase DsbC;

*recJ*: single-stranded-DNA-specific exonuclease RecJ;

*prfB*: peptide chain release factor 2; *lysS*: lysine-tRNA ligase;

*idi*: isopentenyl-diphosphate Delta-isomerase;

*nlpD*: murein hydrolase activator NlpD;

*actS*: amidase activator ActS;

*yjdI*: 4Fe-4S mono-cluster protein YjdI;

*orfA*: uncharacterized protein, domain of unknown function (DUF4468) with TBP-like fold;

*orfB* - *orfI*: uncharacterized protein;

*orfJ*: anaerobic benzoate catabolism transcriptional regulator;

*orfK*: uncharacterized protein;

*orfL*: glutathionine S-transferase;

*orfM*: uncharacterized protein;

*orfN*: Metallo-beta-lactamase superfamily;

*orfO*: uncharacterized protein;

*orfP*: uncharacterized protein;

*orfQ*: uncharacterized Fe-S cluster protein YjdI;

*orfR*: MBL fold metallo-hydrolase;

*orfS*: transcriptional regulator;

*orfT*: voltage-gated chloride channel family protein;

*orfU*: NtaA/DmoA family FMN-dependent monooxygenase;

*orfV*: LLM class flavin-dependent oxidoreductase.
