# Supplementary figures and images for: Identification and characterization of a novel chromosomal aminoglycoside 3’-O-phosphotransferase, APH(3′)-Id, from Kluyvera intermedia DW18 isolated from the sewage of an animal farm
Source: Front Microbiol. 2023 Aug 28;14:1224464. doi: 10.3389/fmicb.2023.1224464 (PMC10493288; doi:10.3389/fmicb.2023.1224464)

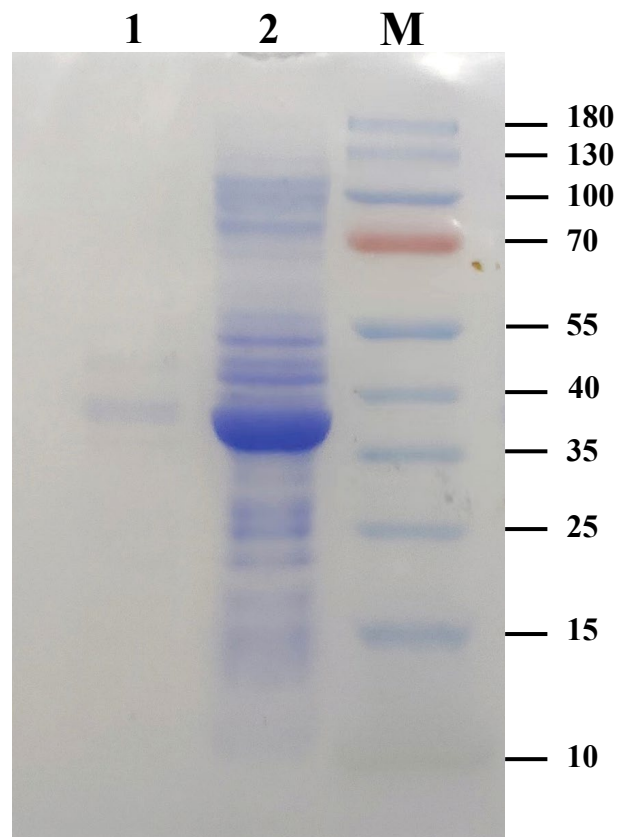

**FT W1 W3 W5 W7 M E1 E2 E3 E4 E5 E6 E7 E8**

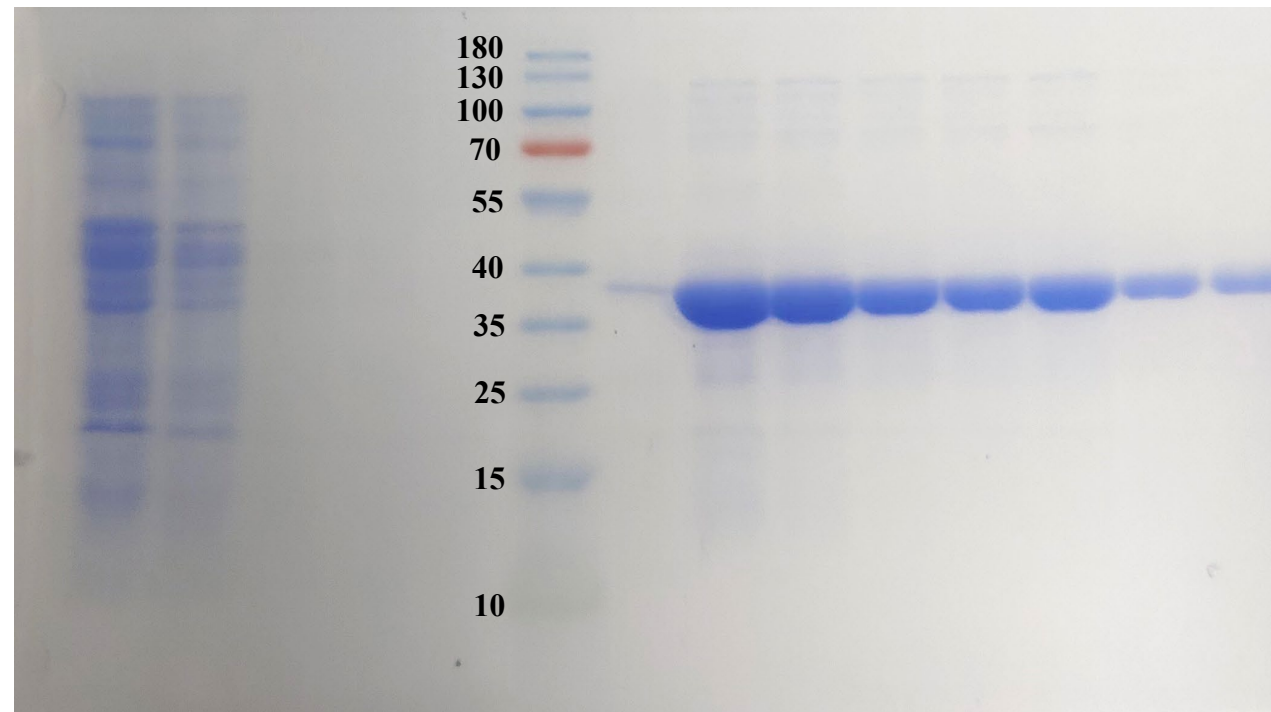

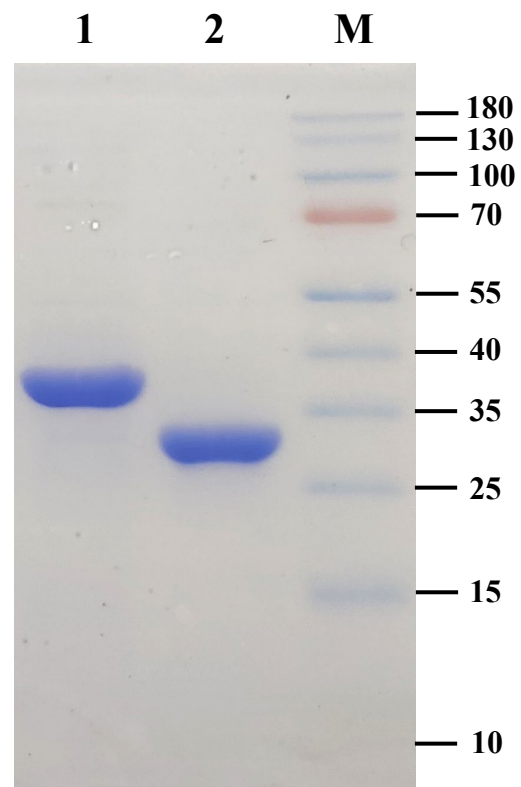

Supplement: Supplementary file 2 [file Data_Sheet_1.PDF]
